# Tracing the Use of the Family Management Framework and Measure: A Scoping Review

**DOI:** 10.1177/1074840721994331

**Published:** 2021-03-20

**Authors:** Kathleen A. Knafl, Janet A. Deatrick, Agatha M. Gallo, Beth Skelton

**Affiliations:** 1 The University of North Carolina at Chapel Hill, USA; 2 University of Pennsylvania, Philadelphia, USA; 3 University of Illinois at Chicago, USA

**Keywords:** family research, family management, scoping review

## Abstract

This article reports the results of a scoping review of research applications of the Family Management Style Framework (FMSF) and the Family Management Measure (FaMM). We identified 32 studies based on the FMSF and 41 studies in which the FaMM was used, 17 of which were based on the FMSF. Both the framework and measure have been used by investigators in multiple countries, with most applications of the FaMM outside the United States. Although the FMSF and FaMM were originally developed for use with families in which there was a child with a chronic physical condition, both have been applied to a broader range of health conditions and to studies focusing on families with an adult member facing a health challenge. Based on our findings, we make recommendations for how researchers can more fully address all aspects of the FMSF.

Published three decades ago (Knafl & Deatrick, 1990), the Family Management Style Framework (FMSF) conceptualized how families with a child with a chronic condition incorporated condition management into daily family life. The FMSF described key elements of family management related to how family members defined their situation, their management behaviors, and the consequences of condition management for family life. Recognizing that these elements might differ across family members, we also pointed to the need to explicate further the elements of family management and identify overarching family management patterns. Research aimed at further developing the framework and identifying patterns of family management was first published in 1996 by Knafl and colleagues.

Building on research on family response to childhood conditions, we continued to refine the framework, publishing two updated versions (Knafl & Deatrick, 2003; Knafl et al., 2012). Based on research evidence, the updated versions of the FMSF added family and family member functioning as outcomes of family management. The most recent version of the framework is displayed in Figure 1, which also highlights the changes made as the framework was developed. These initial conceptualizations of family management were grounded in studies of predominantly North American families in which a child had a chronic physical condition. In 2010 Janice Bell, the editor of the *Journal of Family Nursing* (JFN), invited the submission of manuscripts for a special issue addressing “New Directions for the Family Management Style Framework” that extended the framework to conditions and/or sociocultural contexts not included in the initial conceptualization. Published in May 2012, the special issue reported studies focusing on family management of serious and life-threatening conditions in both children (Bousso et al., 2012; Rempel et al., 2012) and adults (Beeber & Zimmerman, 2012; Wiegand, 2012), studies completed outside North America (Bousso et al., 2012), and a study of adolescents’ perspectives of family management of chronic conditions (Wollenhaupt et al., 2012).

**Figure 1.**
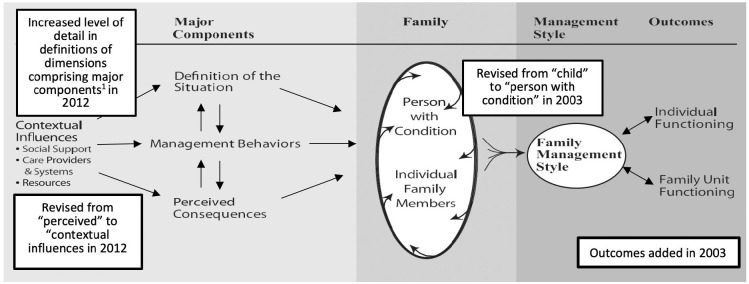
Family Management Style Framework. ^a^Major Components—Definition of the Situation (child identity, view of condition, management mindset, parental mutuality); Management Behaviors (parenting philosophy, management approach); Perceived Consequences (family focus, future expectations).

In the absence of a measure of family management, early studies applying the FMSF were largely qualitative. We recognized that to examine the association between family management and family and family member functioning, a measure of family management was needed. Funding from the National Institute of Nursing Research supported the work to develop a family management measure. A questionnaire was created with items based on the FMSF; the measure’s psychometric properties were assessed from a study of more than 500 parents with children with varied non-life-threatening chronic physical conditions. Publication of the Family Management Measure (FaMM) included its methodological development and provided evidence of its reliability and validity (Knafl et al., 2011). The measure comprised six separately scored scales (Child Daily Life, Condition Management Ability, Condition Management Effort, Family Life Difficulty, Parental Mutuality, and View of Family Impact) with a total of 53 Likert-type items. Researchers have the option of using all or a subset of the scales. There is no summary score because each scale measures a different aspect of family management. The instructions to the FaMM define family as “those living in your household that you think of as family.” The publication of the FaMM set the stage for subsequent quantitative studies, including investigations directed to examining the relationship between family management and child and family outcomes, as well as identification of patterns of family management.

Although we were aware of ongoing applications of the FMSF and FaMM, we had not systematically tracked the extent of their use. We recognized that such tracking would be useful in evaluating the applicability of both, thereby informing study design and decision-making of future investigators. Our intent in this review was to determine how the FMSF and FaMM have been used in research by examining the aims, sample, and design of published research reports.

We conducted a scoping review of published studies to assess the extent and ways in which the FMSF and FaMM had been used. Following guidelines recommended by Arksey and O’Malley (2005), we identified and screened research reports for inclusion in the sample, extracted the information from the reports needed to address the purpose of the review, and collated and summarized the results. With support from a research librarian based at the first author’s university, we identified articles citing one or more versions of the FMSF and/or the FaMM published through June 30, 2019. Articles were identified through citation searches of the Scopus, Web of Science, and Google Scholar databases. Following identification of all articles citing the FMSF and/or the FaMM, the first author reviewed articles to differentiate research from nonresearch applications of the FMSF and FaMM. Research applications were defined as those in which the FMSF provided the conceptual underpinnings of a research study or secondary analysis or the FaMM was used in the study. In some articles, the framework or measure was cited in a background or discussion section but was not applied in the research being reported. Other nonresearch applications included citations in a review article or publications focusing on theory or methods. Other than tallying the number of such citations, these reports were excluded from further analysis. The co-authors reviewed the research versus nonresearch categorization, with any disagreements resolved through discussion to reach consensus.

The following information was extracted from each study: country where data were collected, study aims, sample (number of families; family members participating), condition(s) included in the sample, and design. For studies based on the FMSF, which aspects of the FMSF (contextual influences, components/dimensions, management style, outcomes) were addressed in the research were noted. For studies using the FaMM, we also noted the conceptual underpinnings and summarized how the measure was used in the study (e.g., independent versus dependent variable) and any data provided on the reliability of the six FaMM scales. Four summaries were completed, one for each of the three versions of the FMSF and one for the FaMM.

The first three authors divided the work of extracting the data from the research reports. As a quality check, the fourth author independently extracted the same information from every fifth article. These checks revealed we were thorough and accurate in the data extractions and only minor corrections were made to the initial extractions.

The analysis was straightforward and included simple listings and counts (e.g., countries where data were collected, conditions studied) and categorization of certain aspects of the study (e.g., aims, design, use of FaMM). The first author did the initial categorizations, which were then reviewed by the co-authors. Any disagreements were resolved through discussion to reach consensus. If more than one version of FMSF was cited in the research report, only the most recent version was counted in the tally.

## Results

### Final Sample

In Table 1, the breakdown of the 262 published articles citing one or more versions of the FMSF or citing the FaMM is summarized. The FMSF was named as the conceptual grounding in 32 studies and the FaMM was used in 41 studies, 17 of which were based on the FMSF. Each study was counted in one tally only to avoid artificially inflating the number of studies based on the FMSF. Studies using the FaMM and citing the FMSF were tallied under the FaMM. Both the FMSF and the FaMM also were cited in nonresearch articles and research reports where they were not applied but were referenced in the background or discussion sections of the article. These were categorized as “Other Citations.” Eight reports citing the FaMM addressed efforts to assess or adapt it for use with non-English samples. In contrast to the tally for “Published Research Based on FMSF or FaMM,” when reports in the “Other” category included citations to multiple versions of the FMSF, each of these citations was tallied. The rationale for this was that authors were using the citations to support their work, and thought it necessary, in some cases, to cite multiple versions.

**Table 1.** Number of Reports Citing the FMSF or the FaMM.

Citation	Published research based on FMSF or FaMM Number of studies/number of reports	Other citations to FMSF or FaMM
FMSF 1990	8/8	59
FMSF 2003	16/23	46
FMSF 2012	8/10	34
FaMM	41/44	38
Total	73/85	177

*Note.* Each study was counted in one tally only. If more than one version of FMSF was cited, only the most recent version was counted in the tally. Studies using the FaMM and citing the FMSF were tallied under the FaMM. FMSF = Family Management Style Framework; FaMM = Family Management Measure.

### FMSF

As summarized in Table 2, study participants for most (*n* = 26/81%) of the 32 studies based on the FMSF were recruited in the United States, although investigators from Brazil, Thailand, and Canada also published study results. The research aims of the studies citing the FMSF were most often descriptive or exploratory such as an early study by Williams (1995) describing the problems and management behaviors used by mothers of daughters with precocious puberty or Turner syndrome. More recently, Estrem and colleagues (2017) applied the FMSF to a study of family management of children’s feeding problems and SanGiacomo and colleagues (2019) described the management challenges reported by mothers of childhood brain tumor survivors. In four studies (Conlon et al., 2008; Knafl et al., 1996; McCarthy & Gallo, 1992; Obrecht et al., 1992), investigators described changes in family management over time. One investigator (Johnson et al., 2014) used the framework to support development of a social script iPad application for children with autism.

**Table 2.** Overview of Studies Conceptually Grounded in FMSF.

Study	Country where data were collected	Aims	Sample (families/family members)	Condition(s) studied	Design	Aspects of FMSF addressed in study^a^
1990 Version of FMSF						
Edwards-Beckett & Cedargren (1995)	USA	To describe parents’ perception contextual influences and their level of supportiveness	30 families (30 mothers, 27 fathers)	Myelomeningocele	Qualitative description	• Contextual influences (derived from data)
Gallo (1990)	USA	To describe a family with a child with diabetes from the individual members’ and family’s point of view	One family (mother, father, child, sibling)	Type 1 diabetes	Qualitative case study	• Contextual influences (resources) • Components/dimensions of family management • Management style
Knafl et al. (1996)	USA	To identify styles of family response to childhood chronic illness and explore their relationship to family and family member functioning	63 (62 mothers, 53 fathers, 66 children 7–14 y/o [three families with two ill children], 28 siblings)	Multiple, non-life-threatening chronic conditions	Longitudinal qualitative description	• Contextual influences (resources) • Components/dimensions of family management • Management styles
Krouse (2002)	USA	To identify, describe, and provide a theoretical analysis family management of breastfeeding a low birth weight infant	13 families (13 mothers)	Low birth weight	Longitudinal qualitative description	• Contextual influences (social support) • Components/dimensions of family management • Management styles
McCarthy & Gallo (1992)	USA	To describe family management of Type 1 diabetes and compare responses from the individual family members	One family (mother, father, child with condition, two siblings)	Type 1 diabetes	Longitudinal qualitative case study	• Contextual influences (social support, resources) • Components/dimensions of family management • Management styles
Murphy (1990)	USA	To illustrate three management styles adopted by couples following the birth of a high-risk infant	20 families (20 mothers, 20 fathers)	High-risk infants	Grounded theory	• Components/dimensions of family management • Management styles
Obrecht et al. (1992)	USA	To describe how a family with a child with end-stage renal disease defined and managed their situation	One family (mother, father, child with condition, sibling)	End-stage renal disease	Longitudinal case study	• Components/dimensions of family management • Management styles
Williams (1995)	USA	To identify the problems and management behaviors used by mothers in attempting to solve problems in school, peer, and family life for their daughters with precocious puberty or Turner syndrome	12 families (12 mothers)	Precocious puberty and Turner syndrome	Qualitative description	• Contextual influences (care providers and systems) • Components/dimensions of family management • Management styles
2003 Version of the FMSF						
Athaseri et al. (2008)	Thailand	To describe (a) definition, (b) management behaviors, and (c) perceived consequences of having a child with Type 1 Diabetes.	22 families (22 mothers)	Type 1 diabetes	Qualitative descriptive	• Components/dimensions of family management
Bingham & Haberman (2006)	USA	To describe how spirituality assists people with Parkinson's Disease and their families in defining and managing the day-to-day experience of the disease	27 dyads—24 husband/wife; three parent/child	Parkinson’s disease	Qualitative descriptive	• Contextual influences (spirituality)
Bousso et al. (2012)	Brazil	To explore how families define and manage their life when they have a child or adolescent undergoing palliative home care	14 family members (11 mothers, one father, one aunt, one grandmother)	Children attending an outpatient palliative care unit	Qualitative descriptive	• Components/dimensions of family management
Conlon et al. (2008)	USA	To describe changes in family management following treatment	71 families (51 mothers, 20 fathers)	Attention deficit hyperactivity disorder	Secondary analysis of archival data comparing family management before and after treatment	• Management styles
Gallo et al. (2005, 2009)	USA	To identify patterns of information management and explore their relationship to individual and family characteristics and functioning	86 families (83 mothers, 53 fathers, six others)	Varied single gene conditions	Mixed methods	• Contextual influences (resources) • Components/dimensions of family management • Family information management styles • Outcomes (parent, family)
Hopkins & Gallo (2012)	USA	To describe mothers’ perception of their children’s school life within the context of overall family management	41 families (41 mothers)	Sickle cell anemia, cystic fibrosis	Qualitative descriptive; secondary analysis	• Contextual influences (care providers and systems) • Components/dimensions of family management
Johnson et al. (2014)	USA	To examine the effectiveness of the social script intervention, “Going to Imaging” application (iPad app)	32 parent–child dyads	Autism spectrum disorder	Pilot feasibility study for randomized control trial	Not specified
Knafl et al. (2010)	USA	To identify the parents’ perceptions of normalization and the meaning attributed to its presence or absence	28 families (28 mothers, 20 fathers)	Varied single gene conditions	Qualitative description; secondary analysis	• Components/dimensions of family management
Mendes-Castillo et al. (2012); Mendes-Castillo, Bousso, & Silva, 2014); Mendes-Castillo, Bousso, Ichikawa, et al., 2014)	Brazil	To understand the family management of childhood liver transplantation	Eight families (eight mothers, one father)	Liver transplantation	Qualitative description	• Component/dimensions of family management
Misko & Bousso (2007)	Brazil	To understand family management of cancer at home and decision-making related to seeking emergency care	Six families (six mothers)	Cancer	Qualitative description	• Components/dimensions of family management
Rempel et al. (2012)	Canada	To examine family management hypoplastic left heart syndrome from diagnosis through the early period of home care	24 families (24 mothers, 17 fathers)	Hypoplastic left heart	Qualitative description; secondary analysis	• Components/dimensions of family management • Family management styles
Toly et al. (2012a, 2012b); Toly & Musil (2015)	USA	To examine the interrelationships between normalization, maternal depression, level of child’s technological dependence and illness severity, family functioning, and sociodemographic characteristics	102 families (102 mothers)	Technology dependent children living at home	Hypothesis testing; longitudinal	Not specified
Van Riper (2005)	USA	To explore the family experience of genetic testing	Two families (Case 1—husband, two daughters; Case 2—three adult sisters)	Huntington disease (Case 1) Breast cancer gene (Case 2)	Case study	• Components/dimensions of family management
Wiegand et al. (2008); Wiegand (2012)	USA	To describe family management after the death of a family member who had life-sustaining therapy withdrawn	19 families (56 family members)	Unexpected life-threatening illness of injury	Phenomenology	• Components/dimensions of family management • Family management styles
Wirattanapokin et al. (2013)	Thailand	To explore patterns of self-management among adolescents with prediabetes or Type 2 diabetes	16 families (16 adolescents)	Prediabetes and Type 2 diabetes	Grounded theory	• Contextual influences (social support, resources) • Components/dimensions of family management • Family management styles
Wollenhaupt et al. (2012)	USA	To uncover the family management perspectives of adolescents with spina bifida	25 families (25 adolescents 12–21 y/o)	Spina bifida	Qualitative descriptive; secondary analysis	• Components/dimensions of family management
Young (2013)	USA	To explore how families with children at home manage 4–12 months after an adult family member had been discharged from the hospital following a bone marrow transplantation	15 families (15 patients, 14 adult significant others)	Bone marrow transplant	Qualitative description	• Components/dimensions of family management
2012 Version of FMSF						
Beacham & Deatrick (2015)	USA	To describe children’s perceptions of family management	32 families (32 children, 32 parents)	Multiple non-life-threatening chronic conditions	Qualitative description	• Components/dimensions of family management
Cody et al. (2018)	USA	To describe the perspectives (view of the illness, role in future management, and long-term consequences on individual and family functioning) of family members of patients in the intensive care unit who participated in family bedside rounds versus those who did not	19 families (19 family members—unspecified)	Adult hospitalized on Intensive care unit	Qualitative description	• Components/dimensions of family management
Estrem et al. (2017)	USA	To describe parents’ perspectives of their child’s eating and of feeding management and to identify themes of feeding management in the context of everyday family life	Nine families (nine mothers, three fathers)	Feeding problem	Qualitative description	• Components/dimensions of family management
Fleming, Knafl, & Van Riper (2017)	USA	To examine, based on the gender of the child, the varying family experiences of having a child with congenital adrenal hyperplasia	Nine families (nine mothers, seven fathers)	Congenital adrenal hyperplasia	Mixed methods analysis based on qualitative interviews	• Components/dimensions of family management
Koplow et al. (2015a, 2015b)	USA	To examine the experiences of family members in the nursing home placement and care of an older family member	10 families (10 primary caregivers; varied family relationships)	Older family member in a nursing home	Longitudinal qualitative description	• Contextual influences (social support) • Components/dimensions of family management
Raymond et al. (2017)	USA	To describe parents’ perspectives on managing the care of their adult child with serious mental illness; functioning	30 families (30 parents; predominantly mothers)	Serious mental illness	Qualitative description	• Contextual influences (care providers and systems) • Components of family management
SanGiacomo et al. (2019)	USA	To describe the family management challenges facing families of childhood brain tumors	45 families (45 mothers)	Brain tumor survivor	Mixed methods; secondary analysis data from qualitative interviews	• Component/dimensions of family management • Family management styles

*Note.* FMSF = Family Management Style Framework.

a Sociocultural context listed only if included in the analysis beyond describing demographic characteristics of sample. ^b^Research study was the unit of analysis.

Beginning with the 1990 version of the FMSF, investigators were extending the framework to the study of health challenges not included in its original development. For example, Krouse (2002) applied it in a study of family management of feeding low birthweight infants; Van Riper (2005) explored the family experience of genetic testing; and Raymond and colleagues (2017) described parents’ perspectives on managing the care of their adult child with serious mental illness.

Consistent with their descriptive or exploratory intent, most investigators described their study design as qualitative, with six being longitudinal. In most studies (*n* = 25/78%), investigators addressed a single condition (e.g., Type 2 diabetes, end-stage renal disease) or group of related conditions (e.g., cancer, serious mental illness). In eight studies, researchers focused on a health-related challenge not linked to a specific condition (e.g., technology dependence, feeding problem). The number of families included in the samples ranged from case studies of one or two families to (Gallo, 1990; McCarthy & Gallo, 1992; Obrecht et al., 1992; Van Riper, 2005) to Toly and colleagues’ (Toly et al., 2012a, 2012b; Toly & Musil, 2015) study of more than 100 families of children with technology dependence. About one half (51%) of studies enrolled 20 families or less and only four studies enrolled participants from 50 or more families.

Family members included in the sample varied across studies. In nine studies, sample inclusion criteria required participation by multiple family members, with five focusing on dyads within the family. For example, both Beacham and Deatrick (2015) and Johnson and colleagues (2014) recruited child–parent dyads; Murphy (1990) included mother–father dyads; and Young (2013) recruited adult patients who had received a transplant and an adult family member. Investigators reporting family case studies included data from an even broader range of family members. McCarthy and Gallo (1992) purposely selected a family for their case study of family management that included interview data from the mother, father, child with the chronic condition, and one sibling. Investigators in nine studies offered family members the option of participation by multiple family members, but this was not a requirement for inclusion in the sample. In other studies, a family member fulfilling a specified family role (e.g., primary caregiver) was recruited and the family designated who would represent them in the sample. When families were given the option of participation by multiple family members or asked to designate a family participant, the final sample predominantly comprised mothers. In seven studies, inclusion criteria specified mothers.

We also examined the extent to which investigators studied all or selected aspects of the framework. Across the 32 studies based on the FMSF, Gallo and colleagues’ (2005, 2009) study of family information management was the only one that addressed all four aspects of the framework (Contextual Influences, Major Components, Management Styles, and Outcomes). More often researchers studied selected aspects of the framework. Investigators addressed a single aspect of the FMSF in 14 studies and two or three aspects in 15 studies. Of the 14 studies focusing on a single aspect of the framework, most (*n* = 11) focused on describing how the six dimensions of family management were reflected in their sample. For example, Estrem and colleagues (2017) used the dimensions of the framework to describe parents’ efforts to manage a child’s feeding problems and Misko and Bousso (2007) used them to describe family management of a child’s cancer at home. In 12 of the 32 studies, investigators went beyond describing how the FMSF dimensions were reflected in their sample to identify styles of family management based on the FMSF dimensions, with the styles varying across samples. For example, Rempel and colleagues (2012) described changing patterns of family management in parents of children who were surgically treated for a life-threatening cardiac condition. Investigators in nine studies examined parents’ perceptions of the link between the contextual influences included in the framework and family management.

### FaMM

In Table 3, the characteristics of studies using the FaMM are summarized, and in Tables 4 and 5, reports of the internal consistency reliability (ICR) for FaMM scales are presented. Researchers from 11 countries have reported using the FaMM. In contrast to the FMSF where U.S.-based samples predominated, the majority (*n* = 25/61%) of studies using the FaMM were conducted outside the United States.

**Table 3.** Overview of Studies Using the FaMM.

Article	Country where data collected	Aims (as stated by authors)	Conceptual framework	Sample design and no. of families and family members	Sample—condition	Design	How family management scales used	Aspects of FMSF addressed in study^a,b^
Caples et al. (2018)	Ireland	To examine families of children with Down syndrome residing in Ireland adapt to their child’s diagnosis	Resiliency Model of Family Stress, Adjustment, and Adaptation	95 families (79 mothers, 16 fathers)	Down syndrome	Mixed methods	Predictor—FaMM scales as predictor of family adaptation	Not applicable
Choi (2015)	Korea	To examine factors influencing family adaptation in Korean families with a child with Down syndrome	Resiliency Model of Family Stress, Adjustment, and Adaptation	147 families (147 parents—unspecified)	Down syndrome	Mixed methods	Predictor—FaMM scales as predictor of family adaptation	Not applicable
Choi & Van Riper (2014)	Korea	To explore Korean mothers’ perceptions of their typically developing children when there is a child with Down syndrome in the family	Resiliency Model of Family Stress, Adjustment, and Adaptation	105 families (105 mothers)	Down syndrome	Descriptive cross-sectional survey	Predictor—FaMM scales as predictor of sibling adaptation	Not applicable
Close et al. (2016)	USA	To describe family management challenges for parents who have sons with Klinefelter Syndrome	FMSF	40 families (33 mothers, seven fathers)	Klinefelter syndrome	Mixed method	Outcome—Influence of parents’ age on family management	• Contextual influence (care providers and systems) • Components/dimensions of family management • Outcomes (parent and family)
Deatrick et al. (2014)	USA	To test a hypothesized model of caregiver competence.	Model based on literature and Raina et al., theoretical stress process model	186 families (186 mothers)	Brain tumor survivors	Cross-sectional telephone survey	Predictor—Management Ability scale as predictor caregiver competence	Not applicable
Deatrick et al. (2018)	USA	To develop a typology of family management patterns of brain tumor survivors and examine the relationship between pattern membership and family functioning and health-related quality of life	FMSF	186 families (186 mothers, 134 survivors)	Brain tumor survivors	Mixed methods	Descriptive—patterns of family management based on scales Correlate—patterns and family function and mother and survivor health-related quality of life	• Contextual influences (resources) • Components/dimensions of family management • Family management styles • Outcome (survivor, mother, and family functioning)
Duangdech et al. (2017)	Thailand	To validate a causal model of factors contributing to the health status of children with cerebral palsy in Thailand	Resiliency Model of Family Stress, Adjustment, and Adaptation and the literature	208 families (131 mothers; identity of other family members unspecified)	Cerebral palsy	Cross-sectional correlational	Mediator—Family management as a mediator of the relationship between social support, family hardiness, access to care, illness severity and of child’s health status	Not applicable
Fleming, Knafl, Knafl, & Van Riper (2017)	USA	To describe adrenal crisis events in children with congenital adrenal hyperplasia and examine the relationship between parents’ perceived management ability and the impact of the condition on family life	FMSF	68 families (60 mother, 15 fathers, two grandmothers)	Congenital adrenal hyperplasia	Mixed methods	Correlate—relationship between Management Ability and View Family Impact	• Components/dimensions of family management • Outcome (family functioning)
Geense et al. (2018)	Netherlands	To identify outcome measures and assess potential effectiveness, and design issues for a web-based intervention for parents of children with kidney disease	Not reported	85 families (83 mothers; 50 fathers)	Chronic kidney disease	Randomized controlled trial feasibility study	Outcome—Family management outcome of intervention	Not applicable
Gibson-Young et al. (2014)	USA	To examine the relationships between maternal caregiver perceptions of family management and asthma morbidity	FMSF	101 families (101 mothers)	Asthma	Cross-sectional survey	Correlate—Management Ability and Effort scales and asthma morbidity	• Components/dimensions of family management • Outcome (ill child)
Hackworth et al. (2013)	Australia	To describe the protocol for a study evaluating the efficacy of an online adolescent and parenting intervention	Not reported	120 families (120 adolescents, 120 parents or guardians)	Type 1 diabetes	Randomized control trial	Outcome—Family Management outcome of intervention	Not applicable
Hickey et al. (2018)	Australia	To test the efficacy of a family intervention in promoting early adaptation to a child’s acquired brain injury	Not reported	47 families (38 mothers, 29 fathers, four siblings, one grandmother) Baseline usual care (18 mothers, 11 fathers, one sibling)	Brain injury	Randomized control trial	Outcome—Family management outcome of intervention	Not applicable
Hobbie et al. (2010)	USA	To determine the educational needs of parents as their children completed cancer treatment and assess the feasibility of measuring parental needs, anxiety, and family management as treatment ends	Not reported	15 families (15 caregivers unspecified)	Cancer	Descriptive, cross-sectional	Descriptive—Family management following treatment	Not applicable
Hsiao (2014); Hsiao & Van Riper (2011)	Taiwan	To explore the relationships among family demographics, family demands family appraisal, and individual and family adaption	Resiliency Model of Family Stress, Adjustment, and Adaptation	83 families (80 mothers, 75 fathers)	Down syndrome	Cross-sectional survey	Correlate—Family Management scales and family adaptation	Not applicable
Im et al. (2019)	Korea	To examine the relationship between parenting stress and quality of life in children with epilepsy and the mediating effect of family management	FMSF	93 families (90 mothers, three fathers)	Epilepsy	Cross-sectional survey design	Mediator—Family Management as mediator of relationship of parental stress and child quality of life	• Components/dimension of family management
D. H. Kim & Im (2015)	Korea	To examine the influence of family management on psychosocial problems of childhood cancer survivors in Korea	FMSF	158 families (149 mothers, five fathers, four grandparents)	Cancer	Cross-sectional survey	Correlate—Family Management scales and child’s psychosocial problems	• Contextual influences (resources) • Components/dimensions of family management • Outcomes (survivor functioning)
I. Kim et al. (2016)	USA	To examine the relationships between child behavior problems and mothers’ depressive symptoms	FMSF	234 families (234 mothers)	Autism	Cross-sectional survey	Mediator—Family Management as a mediator of relationship between child’s behavior problems and mother’s depressive symptoms	• Components/dimensions of family management • Outcomes (maternal functioning)
Knafl et al. (2013)	USA	Identify patterns of family management and examine their relationship to child and family functioning	FMSF	414 families (414 mothers, 161 fathers)	Multiple chronic physical conditions	Cross-sectional survey	Descriptive—Patterns comprised Family Management scales	• Components/dimensions of family management • Family management styles • Outcomes (child and family functioning)
Lerret et al. (2015)	USA	To examine parents’ readiness of hospital discharge and its relationship to postdischarge coping, family impact, adherence to medications, and follow-up, and utilization of following hospital discharge.	Meleis Transitions Theory	51 families (44 females, seven males)	Solid organ transplant	Prospective, longitudinal, correlational design	Outcome—Influence of discharge readiness Condition Management Effort scale	Not applicable
Lindsey (2016)	USA	To examine in families that have a child with a special health care needs, what are the effects of respite care through a therapeutic summer day camp program on family management	FMSF	22 families (20 mothers, one father, one grandmother)	Children with special health care needs	Mixed method	Outcome—respite camp program leading to better family Management	• Components/dimensions of family management
Lohan et al. (2017)	Australia		Literature-based model	186 families (178 mothers, eight fathers)	Type 1 diabetes	Cross-sectional survey	Correlate—Relationship of Management Effort to parents’ diabetes self-efficacy	Not applicable
Mendes et al. (2016)	Portugal	To compare family functioning and parents’ and children’s adaptation to chronic conditions	Socioecological framework of adaptation	263 families (227 mothers, 36 fathers)	Asthma, obesity, epilepsy, diabetes	Cross-sectional survey	Mediator—Family Life Difficulty and Parental Mutuality as mediator of relationship between family cohesion and family member adaptation	Not applicable
Mendes et al. (2017)	Portugal	To examine the moderating role of parents’ social comparison orientation in the associations between family management and children’s health-related quality of life and perceived stigma	Social Comparison Orientation	201 families (201 primary caregivers, 201 children)	Epilepsy	Cross-sectional survey	Predictor—Child Daily Life and Family Life Difficulty as predictor of child’s health-related quality of life and perceived stigma	Not applicable
Muscara et al. (2015); Rayner et al. (2016)	Australia	To examine the prevalence and trajectory of parent psychosocial distress, identify demographic, psychosocial and illness-related predictors of parent psychosocial distress over time, and examine the relationship between parent psychosocial distress and child psychological well-being	Kazak’s Pediatric Medical Traumatic Stress Model	192 families (180 mothers, 76 fathers)	Child with a life-threatening illness or injury	Prospective longitudinal survey	Moderator—FaMM used as a measure of the illness experience, which was described as a moderator parents’ psychosocial distress	Not applicable
Quast et al. (2018)	USA	To examine the prospective influence of family functioning on survivor quality of life months later	Paterson and Drotar’s theoretical model of childhood cancer survivorship	35 families (35 mothers)	Brain tumor survivors	Longitudinal survey	Correlate—Family Life Difficulty and survivor quality of life	Not applicable
Quast et al. (2016)	USA	To describe the physical and psychosocial health-related quality of life of parents of brain tumor survivors and variables associated with parents’ quality of life	Raina’s conceptual model of caregiving	50 families (48 mothers, two fathers)	Brain tumor survivors	Longitudinal survey	Correlate—Relationship of Condition Management Effort and Condition Management Ability to parents’ quality of life	Not applicable
Rearick et al. (2011)	USA	To explore the experiences of parents and children newly diagnosed with Type 2 diabetes with peer social support following an intervention to enhance support	Adapted version of Ireys et al.’s (2001) Social Support Framework	11 families (8 mothers, 3 fathers)	Type 1 diabetes	Mixed methods	Descriptive—FaMM scales used to describe parents’ experiences	Not applicable
Salvador et al. (2019a, 2019b)	Portugal	To examine the contribution of individual and family factors to psychological well-being of parents of children and adolescents diagnosed with cancer.	FMSF Social ecological Theory	205 families (177 mothers, 28 fathers)	Cancer—in treatment or completed treatment in past 5 years	Cross-sectional survey	Correlate—Relationship of Family Life Difficulty, Parental Mutuality to parenting satisfaction, parental anxiety and depression	Not applicable
Sananreangsak et al. (2012)	Thailand	To examine variables contributing to parents’ medical, role, and psychosocial management behaviors	FMSF	88 families (69 mothers, 16 fathers, three unknown)	Thalassemia	Cross-sectional survey	Predictor—FaMM scales as predictor of medical management, role management, and psychosocial management	• Component/dimensions of family management • Outcome (family functioning)
Sheng et al. (2018)	China	To explore the relationships between family management, self-management, and transition readiness	Hypothesized model based on the literature	268 families (268 youth, 181 mothers, 46 fathers, 41 grandparents)	Diabetes, rheumatic disease, renal disease	Cross-sectional survey	Predictor—family management as a predictor of youth self-management, transition readiness, and quality of life	Not applicable
Son, Kim, et al. (2018)	Korea	To identify the variables that affect family management of childhood atopic dermatitis	Bandura’s Self-Efficacy Theory	168 families (168 mothers)	Atopic dermatitis	Cross-sectional survey	Outcome—Influence of disease severity on Condition Management Effort Predictor—Condition Management Effort as a predictor of Condition Management Ability Effort	Not applicable
Son, Song, et al. (2018)	Korea	This study aimed to identify the effects of the mother-medical staff partnership on mothers’ condition management ability	None reported	109 families (109 mothers)	Chronic allergic diseases	Cross-sectional survey	Outcome—Influence of mother-medical staff partnership on Condition Management Ability	Not applicable
Sullivan-Bolyai et al. (2016)	USA	To explore feasibility/ability to recruit and conduct a two-arm trial on reeducation, collaboration, and social support	FMSF	22 families (22 mothers, 22 youth)	Type 2 diabetes	Randomized controlled trial Feasibility study	Outcome—Family management as an outcome of trial	• Contextual influences (resources) • Components/dimensions of family management
Swallow et al. (2012)	England	To describe the protocol for an online parent information and support package for home-based care	None reported	80 families (80 primary caregivers—unspecified)	Kidney disease	Randomized controlled trial feasibility study	Outcome—Family management as an outcome of trial	Not applicable
Van Riper et al. (2018)	USA	To compare family management of children with a chronic physical condition to family management of a child with Down syndrome using data from two studies	FMSF Resiliency Model of Family Stress and Adaptation	Chronic physical conditions—412 families (253 mothers, 159 fathers) Down syndrome − 483 families (427 mothers, 56 fathers)	Multiple chronic physical conditions Down syndrome	Secondary analysis of data from two cross-sectional survey	Descriptive—Comparison of family management in two groups	• Contextual influences (resources) • Components/dimensions of family management
Verchota & Sawin (2016)	USA	To examine the relationship of key individual and family self-management theory, context, and process variables on proximal and distal outcomes in adolescents with Type 1 diabetes	Family Self-Management Theory	103 families (103 parent-adolescent dyads)	Type 1 diabetes	Cross-sectional survey	Correlate—Relationship of Family Life Difficulty to self-management behaviors of adolescents	Not applicable
Weissheimer et al. (2018)	Brazil	To investigate the relationship of family management to socio-demographic and physical dependence aspects of children and adolescents with neurological disorders	FMSF	141 families (117 mothers, 19 fathers, five other family members)	Neurologic conditions	Cross-sectional survey	Correlate—Relationship of all FaMM scales to child’s physical dependence and multiple socio-demographic variables	• Contextual influences (resources) • Components/dimensions of family management • Outcome (child)
Zhang et al. (2014)	China	To identify patterns and predictors of family management	FMSF	387 (257 mothers; other family members not specified)	Multiple chronic physical conditions	Cross-sectional survey	Descriptive—Identified patterns of family management based on FaMM scales Outcome—Influence of child and family functioning on management pattern	• Components/dimensions of family management • Family management styles
Zhang, Wei, Han, et al. (2013)	China	To determine the applicability of the Family Management Style Framework for Chinese families with a child who has a chronic condition	FMSF	538 families (primary caregivers—unspecified)	Multiple chronic physical conditions	Cross-sectional survey	Outcome—Influence of contextual variables on family management (calculated as two composite scales—Easy Family Management; Difficult Family Management) Mediator—family management as a mediator of relationship between contextual variables and child and family functioning	• Contextual influences (resources) • Components of family management • Outcome (child and family functioning)
Zhang et al. (2015)	China	To determine the key predictors for each aspect of family management in families with children who have chronic condition	FMSF	399 families (265 mothers, 89 fathers, 45 other family members—unspecified)	Multiple chronic conditions	Cross-sectional survey	Outcome—Child and family characteristics and functioning variables as predictors of FaMM scales	• Contextual influences (resources) • Components/dimensions of family management
Zhang, Wei, Zhang, et al. (2013)	China	To investigate the impact of family management on family functioning of families with chronically ill children	FMSF	618 families (618 primary caregivers—unspecified)	Multiple chronic physical conditions	Cross-sectional survey	Predictor—FaMM scales as predictor of family functioning	• Components/dimensions of family management • Outcome (family functioning)

*Note.* FaMM = Family Management Measure; FMSF = Family Management Framework.

a Contextual influences limited to those included in the FMSF (social support, care providers and systems, and resources) that were included in the analysis beyond describing demographic characteristics of sample. ^b^FaMM scales measure components of Family Management Style Framework.

**Table 4.** Internal Consistency Reliability of Family Management Measure in Studies Reporting a Range of Scores Across Scales.

Study	Scale range
Caples et al. (2018)	>.70 all scales
Choi & Van Riper (2014)	.63–.90 (View of Condition Impact not reported)
Close et al. (2016)	>.68 all scales
Deatrick et al. (2018)	.72–.89
Im et al. (2019)	.65–.89
D. H. Kim & Im (2015)	.69–.90
I. Kim et al. (2016)	.64–.91
Knafl et al. (2013)	>.70 all scales
Zhang, Wei, & Han (2013) ^a^	.62–.84

a Conducted multiple studies reporting same internal consistency reliability range, which was established in first study to determine applicability of the measure to families in China.

**Table 5.** Internal Consistency Reliability of Individual Family Management Scales.

Scale	ICR .70 or better	ICR < 70
Child Daily Life	Hsiao & Van Riper (2011)—.78 Mendes et al. (2017)—.73 Van Riper et al. (2018)—.73–.79^a^	Lerret et al. (2015)—.65
Condition Management Ability	Deatrick et al. (2014)—.74 Fleming, Knafl, Knafl, & Van Riper. (2017)—.81 Hsiao & Van Riper (2011)—.73 Son, Kim, et al. (2018)—.81 Van Riper et al. (2018)—.72–.77	Lerret et al. (2015)—.52 Quast et al. (2016)—.61
Condition Management Effort	Hsiao & Van Riper (2011)—.72 Quast et al. (2016)—.74 Van Riper et al. (2018)—.74–.78	Lerret et al. (2015)—.51 Lohan et al. (2017)—.68 Son, Kim, et al.(2018)—.60
Family Life Difficulty	Hsiao & Van Riper (2011)—.87 Lerret et al. (2015)—.87 (heart transplant subsample) Mendes et al. (2016)—.83 Mendes et al. (2017)—.86 Quast et al. (2018)—.81 Salvador et al. (2019a, 2019b)—.87 Van Riper et al. (2018)—.91–.92	
Parental Mutuality	Hsiao & Van Riper (2011)—.74 Lerret et al. (2015)—.74 Mendes et al. (2016)—.79 Salvador et al. (2019a, 2019b)—.86 Van Riper et al. (2018)—.75–.88	
View Condition Impact	Hsiao & Van Riper (2011)—.74 Van Riper et al. (2018)—.68–.77	Lerret et al. (2015)—.58

*Note.* ICR = internal consistency reliability.

a Range of ICR scores for two groups of parents included in secondary analysis, those with a child with Down syndrome and those with a child with a chronic physical condition.

Seventeen of the 41 studies using the FaMM were based on the FMSF, eight of which were conducted outside the United States. Eighteen studies were based on other frameworks, providing evidence that the FaMM is suitable for use in studies with diverse theoretical underpinnings. Authors of six studies did not report using a framework. Five studies of families of children with Down syndrome undertaken in Ireland (Caples et al., 2018), Korea (Choi, 2015; Choi & Van Riper, 2014), Taiwan (Hsiao, 2014; Hsiao & Van Riper, 2011), and the United States (Van Riper et al., 2018) were based on the Resiliency Model of Family Stress, Adjustment, and Adaptation (McCubbin & McCubbin, 1993). Other examples of using the FaMM in studies not based in the FMSF include Muscara and colleagues’ (2015; Rayner et al., 2016) use of Kazak’s Pediatric Medical Traumatic Stress Model (Kazak et al., 2006) in their study of parents of children with a life-threatening condition and Son, Kim, and colleagues' (2018) use of Bandera’s Self-Efficacy Theory (Bandura, 1997) to examine factors influencing family management in the context of having a child with atopic dermatitis. In two studies, investigators reported using two frameworks, one of which was the FMSF (Salvador et al., 2019a, 2019b; Van Riper et al., 2018). In 30 studies, investigators used all FaMM scales. In the remaining 11 studies, investigators used selected scales.

For the 17 studies based on the FMSF and using the FaMM, we also examined what aspects of the framework investigators studied. Because the FaMM scales were grounded in the FMSF dimensions, all studies addressed this aspect of the framework. Except for Lindsey’s (2016) study of family management following parents’ participation in a respite program and Im and colleagues’ (2019) study of the mediating effect of family management on the relationship between parental stress and child quality of life, all other studies addressed multiple aspects of the framework. Researchers in 11 studies measured outcomes of family management for the person with the condition, parents, and/or the family system and investigators in eight studies examined contextual influences on family management, with most addressing resources.

Across studies, investigators incorporated the FaMM in varying ways. Using correlation or regression analyses, 28 studies focused on examining the relationship between family management as measured by one of more FaMM scales and other family or family member variables. For example, Gibson-Young and colleagues (2014) examined the relationship between maternal perceptions of Condition Management Ability and Condition Management Effort and asthma morbidity in 101 children in the United States. Lohan and colleagues (2017) studied the relationship between parents’ Condition Management Effort and their perceptions of their diabetes self-efficacy. In other studies, investigators examined variables such as parental age (Close et al., 2016) or family functioning (Zhang et al., 2015) as predictors of family management. In seven studies, investigators examined the mediating or moderating effect of FaMM scales. For example, in a study of Korean families in which a child had epilepsy, Im and colleagues (2019) examined the mediating effect of family management on the relationship between parenting stress and the child’s quality of life. The FaMM scales also were used to compare family management of different types of conditions as in Van Riper and colleagues’ (2018) comparison of families of children with Down syndrome to those of children with a chronic physical condition. In three studies (Deatrick et al., 2018; Knafl et al., 2013; Zhang et al., 2014), investigators used cluster analysis of the six FaMM scales to identify patterns of family management.

Across studies, samples varied as to condition, size, and family member participants. The 18 different conditions studied included chronic physical conditions such as Type 1 diabetes and asthma, cancer and other life-threatening conditions, genetically based conditions, and conditions associated with intellectual or developmental disabilities. Most investigators studied a single condition or related conditions (e.g., cancer, neurologic disease), with only seven studies recruiting sample families in which children were diagnosed with different conditions. The number of families included in these samples ranged from 11 to 895, with more than half (*n* = 25; 61%) including more than 100 families. In 29 studies (71%), only one family member participated in the study, with 13 studies explicitly recruiting mothers, six specifying parent or primary caregiver, and the remaining recruiting family members in varied roles. In the latter case, mothers always made up most of the sample. Investigators in four studies recruited parent–child dyads (Hackworth et al., 2013; Sheng et al., 2018; Sullivan-Bolyai et al., 2016; Verchota & Sawin, 2016). In eight studies, participation by multiple family members was optional.

Investigators varied in the extent to which they reported the ICR of the FaMM scales in their study, with 15 reporting no results or citing the ICR data from the instrument development study (Knafl et al., 2011). In nine studies investigators reported a range of scores across scales (Table 4), with the ICR reaching .70, often considered the cut-off for acceptable reliability (DeVellis, 2003) in three studies and .62 or higher for all scales in the remaining studies. Investigators for 10 studies reported the ICR for each scale used in the study. Regarding individual FaMM scales, Table 5 provides evidence of the reliability of FaMM scales across multiple conditions and sociocultural contexts. The strongest evidence for ICR was for the Family Life Difficulty scale. In seven studies, investigators from different countries (Korea, Portugal, United States) studying different conditions (brain tumor survivorship, cancer, Down syndrome, epilepsy, solid organ transplant, multiple conditions) reported ICR scores ranging from .81 to .92 and no investigator reported an ICR value less than .70. Support for the ICR of Parental Mutuality also was strong with investigators from five studies based on samples representing different countries (Korea, Portugal, United States) and conditions (cancer, Down syndrome, solid organ transplant, multiple conditions) reporting ICR scores greater than .70. For the remaining four scales (Child Daily Life, Condition Management Ability, Condition Management Effort, View of Condition Impact), there were reports of ICR scores both exceeding and falling below .70, though those exceeding predominated and reflected samples representing different countries and conditions. The weakest evidence for support of ICR was for the View of Condition Impact scale. Investigators in two studies (Duangdech et al., 2017; Sananreangsak et al., 2012) incorrectly scored the FaMM by calculating a total single score rather than individual scales scores.

## Discussion

Through this scoping review, we traced the use of FMSF and FaMM and described the frequency and range of research applications across conditions, age groups, and countries. Our review provides evidence of the broad applicability of both the framework and measure and highlights areas of concentrated activity and areas of limited application. The review also provides evidence to guide future research.

Although the framework and measure were developed based on studies of families in the United States with children with non-life-threatening chronic physical conditions not associated with developmental delays, the review provides strong evidence of broader applicability, especially about the types of conditions studied and cultural context. The FMSF and the FaMM have been used in studies of families in which children have a condition that is life-threatening or includes intellectual disability or developmental delay. Both also have been used in studies of survivorship and the aftermath of serious injury, and the FMSF has been used in studies addressing family response to a health challenge facing an adult family member. The FaMM items reference “our child with the condition,” limiting the applicability of the measure for studies in which an adult is the family member facing a health challenge. Although most researchers using the FaMM focused on family management of childhood chronic conditions, we identified several that used the FaMM to study family life in the context of an adult family member who was living with their parents due to cognitive or physical deficits (see, for example, Deatrick et al., 2018; Van Riper et al., 2018).

The review also provides evidence of the cross-cultural applicability of the FMSF and FaMM with the first study based on a sample from outside the United States conducted in Thailand (Athaseri et al., 2008). Cross-cultural applications increased substantially after the publication of the FaMM and reports of its ICR support its applicability in multiple cultural contexts. Not all authors reported the ICR data for their sample. Doing so would help future investigators make informed decisions about the appropriateness of the measure for their intended sample. In addition to the ICR data summarized in Table 4, Van Riper and colleagues’ (2020) analysis of the use of the FaMM with samples of parents of individuals with Down syndrome from 11 countries spanning North and South America, Europe, and Asia provides additional support for the measure’s broad applicability.

The items comprising the FaMM were grounded in the FMSF, and the scales were identified using well-established techniques for instrument development (DeVellis, 2003; Knafl et al., 2011). The scales measure different aspects of family management, with each scale addressing a different underlying latent construct. Despite the FaMM being described as comprising six separate scales with no total score and scoring instructions available on the FaMM website (https://nursing.unc.edu/research/office-of-research-support-and-consultation/family-management-measure/), we found instances of investigators calculating a total score that was then used in their analyses. Such analyses are questionable, and we encourage anyone using the FaMM to adhere to scoring instructions.

Across conditions and cultural contexts, most investigators have focused on examining the FMSF components (Definition of the Situation, Management Behaviors, Perceived Consequences) and their underlying dimensions or the FaMM scales, with less attention directed to identifying patterns of family management, contextual influences, and outcomes. This focus is understandable given the design complexity and resources needed to incorporate multiple aspects of the framework into a single study. However, from the outset the developers maintained that a strength of the framework was the emphasis on identifying patterns of family management based on the configuration of the dimensions of family management across family members. Beginning with the first publication (Knafl & Deatrick, 1990), the framework included influences on family management, and in the first revision (Knafl & Deatrick, 2003), family member and family system outcomes of family management were added.

Although recognizing it may not be feasible to include multiple aspects of the framework in a single study, we encourage investigators focusing on selected aspects of the framework to consider addressing those that have been less studied. For example, contextual influences such as health insurance, access to specialty care, and family income could be included in the demographic and family information respondents often are asked to provide and examined in the analysis as contextual influences on family management. Another possibility would be to add measures addressing social support or health care relationships to examine how these influence family management. The FMSF was based on syntheses of predominantly qualitative research addressing family response to childhood chronic conditions. The contextual influences included in the framework were based on study results reporting what family members identified as factors contributing to the ease or difficulty of family management. Although the contextual influences included in the FMSF reflect issues recognized as social determinants of health that are widely acknowledged as important to understanding structural impediments to families in managing health conditions, there are other factors that are recognized and could be included. Further attention to the social determinants of health also calls for recognition and study of issues such as racism that are now widely acknowledged as important to explaining health disparities (Deatrick, 2017). Although recommending that researchers consider extending their study aims beyond the current focus on the components and dimensions of family management, the body of research addressing these provides an opportunity for undertaking research syntheses of results reporting how the dimensions of family management are reflected across conditions and cultures.

Qualitative data related to the dimensions of family management and scores on the FaMM scales are the building blocks for identifying family management styles, and when investigators have these data, they can take the next step and extend their analysis to identifying different styles. By using analytic approaches such as matrix display of qualitative data reflecting family management dimensions across families and quantitative cluster analyses based on the FaMM scales, investigators can identify styles of family management that can be compared with those previously identified (Deatrick et al., 2018). Although full delineation of management styles, requires a large enough sample to identify subgroups within the sample with similar patterns of family management, even with relatively small samples, investigators can undertake exploratory analyses to detect provisional management styles. By identifying management styles and examining their relationship to family and family member outcomes, investigators will have a better understanding of the management approaches that put families and family members at risk for poor outcomes. Understanding the implications of different management styles also will contribute to the development of tailored interventions aimed at buttressing family management strengths and mitigating problematic aspects.

Sample design presents another opportunity for taking full advantage of the FMSF by including multiple family members in the sample. Beginning with our initial conceptualization (Knafl & Deatrick, 1990), we have emphasized the value of including the perspectives of multiple family members in studies addressing family management. Nonetheless, most investigators applying the framework or using the measure have limited their sample to one family member, most often the mother. The inclusion of multiple family members in the sample provides researchers with the opportunity to undertake conceptually grounded, dyadic, or family system analyses examining the implications of family members having shared versus discrepant or conflicting views of family management (Deatrick et al., 2020).

The focus of both the FMSF and FaMM is family management within a single household and that focus is reflected in the research applications to date. The contribution to condition management of individuals outside the household is incorporated under the Social Network Contextual Influence. Yet to be addressed are those situations where members from multiple households are actively involved in condition management as might be the case when divorced parents have shared custody of a child or when extended family members assume major caregiving responsibilities. Additional studies are needed to examine the unique challenges of multi-household family management and factors supporting and hindering effective, well-coordinated management across households.

Like all scoping reviews, there were limitations to this one. We included only those studies published in English language journals. It is possible that our search missed relevant publications, but we believe we minimized that possibility by working closely with a research librarian to identify relevant citations. The review was limited to research applications because our initial screen of the reports revealed few clinical applications to date. Research reports often make recommendations related to practice applications and these provide a beginning point for clinicians to consider how the FMSF could guide practice or how the FaMM could be incorporated into clinical assessments.

## Conclusion

When we began developing the FMSF in the late 1980s, our objective was to provide researchers with a framework that would guide efforts to advance knowledge of family response to childhood chronic conditions. Building on existing research, we aimed to devise a framework that was applicable to multiple conditions and family structures. We believe this review of research studies supports the conclusion that we achieved that goal. Over the years, others have applied the framework to a much broader spectrum of conditions, populations, and cultural contexts than we originally anticipated. We are grateful for their creative and innovative applications, which have contributed in important ways to extending the scope of the FMSF.

In urging investigators to incorporate multiple aspects of the FMSF into their studies and focus on the less developed aspects of sociocultural context, management styles, and outcomes, we recognize this often requires design trade-offs, especially regarding sampling. Trade-offs typically entail balancing the ideal family research design with the reality of limited resources. For example, a decision to study dyads leads to a decision to study fewer families or a statistical technique requiring a large sample size leads to a decision to recruit only one family member. Considering all aspects of the FMSF when planning a study can help investigators evaluate various options related to their study aims and design and contribute to making well-reasoned decisions about their priorities for advancing knowledge of family management of health-related challenges.
